# A deep LSTM network for the Spanish electricity consumption forecasting

**DOI:** 10.1007/s00521-021-06773-2

**Published:** 2022-02-05

**Authors:** J. F. Torres, F. Martínez-Álvarez, A. Troncoso

**Affiliations:** Data Science and Big Data Lab, Universidad Pablo de Olavide, 41013 Seville, Spain

**Keywords:** Deep learning, Time series forecasting, Electricity demand

## Abstract

Nowadays, electricity is a basic commodity necessary for the well-being of any modern society. Due to the growth in electricity consumption in recent years, mainly in large cities, electricity forecasting is key to the management of an efficient, sustainable and safe smart grid for the consumer. In this work, a deep neural network is proposed to address the electricity consumption forecasting in the short-term, namely, a long short-term memory (LSTM) network due to its ability to deal with sequential data such as time-series data. First, the optimal values for certain hyper-parameters have been obtained by a random search and a metaheuristic, called coronavirus optimization algorithm (CVOA), based on the propagation of the SARS-Cov-2 virus. Then, the optimal LSTM has been applied to predict the electricity demand with 4-h forecast horizon. Results using Spanish electricity data during nine years and half measured with 10-min frequency are presented and discussed. Finally, the performance of the proposed LSTM using random search and the LSTM using CVOA is compared, on the one hand, with that of recently published deep neural networks (such as a deep feed-forward neural network optimized with a grid search) and temporal fusion transformers optimized with a sampling algorithm, and, on the other hand, with traditional machine learning techniques, such as a linear regression, decision trees and tree-based ensemble techniques (gradient-boosted trees and random forest), achieving the smallest prediction error below 1.5%.

## Introduction

Nowadays, electrical energy is one of the main sources of energy in our society. In addition, the demand for electric energy has a growing trend due to great challenges such as the electric vehicle, and new restrictions are emerging related to the use of renewable energy while ensuring a reliable and secure supply. Since electric energy cannot be stored in large quantities, it is extremely important that the amount of electric energy necessary to cover the demand is generated as approximately as possible.

The demand forecasting is often classified as short-term, medium-term and long-term. Short-term forecasting problems involve predicting events only a few hours or days into the future. Medium-term forecasts extend to a few weeks or months and long-term forecasting problems can extend beyond that by few years. The electricity consumption profile for a working day in Spain usually has a valley corresponding to sleeping hours and two demand peaks, a high peak of consumption corresponding to the hours from 08:00 to 09:00 pm and a lower peak of demand corresponding to working hours during the morning. Some days this peak occurring in the morning is divided into two peaks thus obtaining a *camel* type profile.

The electricity demand analysis has traditionally been done by means of classical statistical tools based on time series models [34, 35]. Time series data can be defined as a chronological sequence of observations on a target variable. In the last years, machine learning techniques have been successfully applied for electricity demand forecasting due to its ability to capture complex non-linear relationships in the data [18, 25–27]. However, deep learning techniques are acquiring a great relevance nowadays to solve a large number of applications in multiple areas due to the enhancements in computational capabilities [33, 40, 41]. In particular, specific deep learning models such as Long Short-Term Memory (LSTM) networks have shown its effectiveness to deal with time series [11, 37, 42].

In this work, a deep LSTM neural network along with a hyperparameter optimization is proposed to forecast energy demand for the next 4 h. First, the hyperparameters defining the architecture of the LSTM such as number of hidden layers and units per layer have been optimized along with several important parameters that have a great influence on the performance of the network as the dropout and learning rates. Next, results using electricity demand from Spain for more than nine years measured with 10-min frequency are reported. In addition, the performance of the proposed LSTM is compared to a deep feed-forward neural network and a temporal fusion transformers (TFT) and other recently published forecasting techniques showing a remarkable improvement in the prediction.

The main novelties of this work include the exhaustive analysis of different optimization processes carried out to obtain the best hyper-parameters along with the use of a very novel, architecture such as TFT for comparative purposes. First, an ad hoc estimation process of the learning rate has been carried out through a dynamic adjustment of the learning rate. Subsequently, a joint optimization of all the hyper-parameters, including the learning rate, has been developed through two optimization methods: a random search and a recently published guided metaheuristic, called CVOA, based on the propagation of the COVID [13]. This in-depth analysis of optimization processes has led to really very good results, errors of 1.45%, that improve all previously published results for the prediction of electricity demand in Spain to the best of the authors’ knowledge. Moreover, extensive experimentation has been done to evaluate the performance of the proposed LSTM, comparing with two deep neural networks, a classical deep feed forward and TFT, currently very novel architecture, in addition to traditional machine learning methods. Consequently, both the network architecture defined by the selected hyperparameters and the results obtained along with the extensive comparison justify the novelty and research contributions of this work.

## Related works

This section reviews the most relevant works related to the application of deep learning models to the problem of electricity demand forecasting.

Two recent reviews analyze the topic of deep learning for time series forecasting. The first one provides a theoretical background and an extensive list of applications, categorized by the type of deep learning model [32]. The second one conducts an experimental study comparing the performance of the most popular deep learning architectures, in terms of efficiency and accuracy [8]. In addition, a specific review that emphasizes the application of machine learning techniques to the problem of electricity forecasting can be found in [12].

Different deep learning architectures have been proposed during the last year to address the electricity load forecasting problem. However, deep feedforward neural networks (DFFNN) and deep recurrent neural networks (DRNN) and their variants have been the most successfully used for this purpose.

The DFFNN have been widely used in the literature in order to obtain forecasts in electric markets. The authors in [23] developed a model to predict hourly demand by using the data provided by Korea Electric Power Corporation. The prediction horizon was set to 24 h. The results achieved outperformed a variety of approaches including ARIMA, SNN or DSHW. A grid search was selected as optimization strategy to optimize the weights of a DFFNN in [31]. The method was developed to be applied to multi-output and multi-step time series. Data from the Spanish electricity market were used and the method outperformed linear regression, gradient boost and decision trees and random forest models. Later, the same dataset was analyzed with another DFFNN, but this time optimized with a random search strategy [30]. The authors claimed that, by using this optimization, the learning time is decreased leading to a reduced execution time. They concluded that competitive results in terms of accuracy were produced, generating a smaller number of models. Divina et al. [4] also used a DFFNN to forecast the Spanish electricity consumption. The main novelty of this work lies in the use of a genetic algorithm (GA) to optimize the hyper-parameters of the deep learning model. The approach proposed outperformed several deep learning models with a variety of optimization strategies, an ensemble model composed of regression trees, artificial neural networks and random forests. An ensemble of DFFNN networks was developed by the authors in [21] to forecast time series of general purpose. After that, this strategy has been also used to forecast load demand time series [20]. More recently, Iruela et al. [6] proposed an approach for energy consumption forecasting by using artificial neural networks. As main novelty, the authors simultaneously processed a large amount of data and models thanks to the parallel implementation with TensorFlow and the GPU usage.

Despite the existence of works using other networks, long short-term memory (LSTM) networks are the most successful algorithms applied to forecast electricity consumption. Thus, the work introduced in [3] explored the use of several LSTM configurations for short to medium-term electricity consumption forecasting. A GA was used to determine the optimal number of layers and time lags. France consumption data were used to validate the suitability of the approach. Bedi et al. [1] proposed a framework that analyzed long-term dependencies in the historical data and short-term patterns in segmented data. LSTM was later applied by including a moving window using electricity demand data from India. The model developed outperformed DRNN, artificial neural networks (ANN) and support vector regression (SVR). A case study of electricity forecasting by using the temperature as exogenous variable can be found in [15]. The LSTM network was automatically optimized using a Matlab toolbox. The results were compared to those of autoregressive moving average (ARMA), seasonal autoregressive integrated moving average (SARIMA) and ARMA with exogenous variables (ARMAX) for several prediction horizons in terms of accuracy. Kwon et al. [7] also fed the LSTM network with exogenous variables. The configuration of the hyper-parameters was done through a trial and error method. Two years were used by the power system operator in Korea to evaluate the model, with an error verging on 1.5%. On the contrary, the LSTM introduced in [38] proposed a data dimensionality reduction to decrease the computation cost. The authors designed two groups of experiments to validate the quality of the approach. Comparisons made with ANN, ARMA and autoregressive fractionally integrated moving average (ARFIMA) confirmed the superiority of the proposed method. Finally, the coronavirus optimization algorithm (CVOA) was proposed in [13] and used to optimize the hyper-parameters of a LSTM network. The reported results outperformed a great number of deep learning models hybridized with well-established optimization heuristics. Data from Spanish electricity consumption were used as benchmark. A multi-layer bidirectional RNN based on LSTM and gated recurrent units (GRU) was introduced in [28] to predict electricity consumption. The authors considered separately the peak loads and seasonality and outperformed the results of ANN and SVR. Recently, Pegalajar et al. used three types of RNN to predict the Spanish electricity demand and compared the results to a wide variety of machine learning models, outperforming all of them [17].

Other deep learning-based approaches have also been used in the literature to forecast electricity consumption. Thus, an early work using Elman neural network (ENN) was introduced in [24]. The work used data from Anand Nagar, India, to evaluate the performance of the model in terms of MSE and MPE. It outperformed several methods, including a weather sensitive model and a non weather sensitive model. Additionally, an ENN optimized with a GA was proposed in 2018 [22]. The approach was tested on Spanish data and its performance compared to non-linear ANN with and without exogenous variables. Later, in 2020, another approach based on ENN but adjusted with particle swarm optimization was proposed in [43]. This time the authors evaluated data from eastern Slovakia. The model outperformed other deep learning algorithms in terms of MAE and RMSE. Qian et al. [19] also used ENN but, this time, it was combined with support vector machines (SVM), after having applied principal component analysis. Actual data from a Chinese industrial park were used to assess the quality of the proposal, showing remarkable performance when compared with other methods.

The use of convolutional neural networks (CNN) can be also found in [2, 10] as a useful method to predict power load. In [10], the authors defined new loss functions as main novelty and outperformed results by LSTM, ANN and SVR. In [2], the CNN used a two-dimensional input with historical load data and exogenous variables for both one-step-ahead (15 min) and 96-step-ahead (24 h). Li et al. [9] had previously proposed a CNN-based approach for short-term load forecasting, using data from a large city in China. Some weather time series were also considered to improve the model and the performance was compared with SVM. However, results achieved did not show significant improvement when considering such exogenous variables. The COVID-19 pandemic has changed the consumption patterns and new studies, in this context, are being published. Thus, CNN were also used for short-term load forecasting in [36]. The authors analyzed data from Romania and compared its efficacy with multiple linear regression and the forecasting results by the Romanian transmission system operator. Wu et al. [39] hybridized a CNN by combining it with a GRU. In particular, the GRU module extracted the time sequence data and the CNN module the high-dimensional data. Data from China were used to validate their approach, which outperformed the results of individual GRU, CNN and ANN models. A wide study and comparison of different deep neural networks were made using photovoltaic data from Italy in [14]. Moreover, the performance was evaluated over four different prediction horizons (1 min, 5 min, 30 min and 60 min) and for one-step and multi-step ahead.

Although other exogenous variables such as meteorological variables are included in other studies for the analysis of electricity consumption, as Taylor explained in [29], for very short-term prediction, as is the case of our work, the electricity forecasting is not highly influenced by weather conditions. Hence, the use of a single variable is sufficient, unless abrupt variations appear which should be considered as a special situation and be treated with ad hoc methods.

## Methodology

This section presents the description of the methodology carried out for electricity consumption forecasting. For this purpose, the problem to be solved will be described in Sect. 3.1. Then, the chosen network architecture will be detailed in Sect. 3.2, and finally, the approach used to optimize the different network hyper-parameters will be discussed in Sect. 3.3.

### Problem formulation

This work is framed in a supervised learning problem. In particular, it is a multi-step regression, where the main goal is: given a times series, which can be expressed as $[x_1, x_2, \dots , x_t]$, to find a model *f* based on a historical data window that allows to forecast future values. Formally, this formulation is shown in Equation 1:

1 $$\begin{aligned}{}[x_{t+1}, x_{t+2}, \dots , x_{t+h}] = f(x_{t}, x_{t-1}, \dots , x_{t-(w-1)}) \end{aligned}$$

where *w* is the number of past values used as historical window and *h* is the number of future values to forecast, also called prediction horizon.

### LSTM architecture

The evolution of deep learning has grown exponentially in recent years. There are several types of architectures, which are used depending on the characteristics of the problem to be solved. Convolutional neural networks (CNN) are often used in image processing while recurrent neural networks (RNN) for sequential data, such as time series analysis and forecasting. However, some studies as the review of deep learning architectures for time series forecasting conducted by the authors in [32] have shown the efficiency of several network architectures to solve problems for which they were not initially designed.

In this work, a LSTM architecture is used to forecast electricity consumption time series. This architecture is framed within recurrent networks, whose main characteristic is the capacity to model temporal dependencies of the data. This makes them highly recommended for sequential data problems such as text transcription, audio or time series, due to a certain memory being provided to the network.

**Fig. 1:**
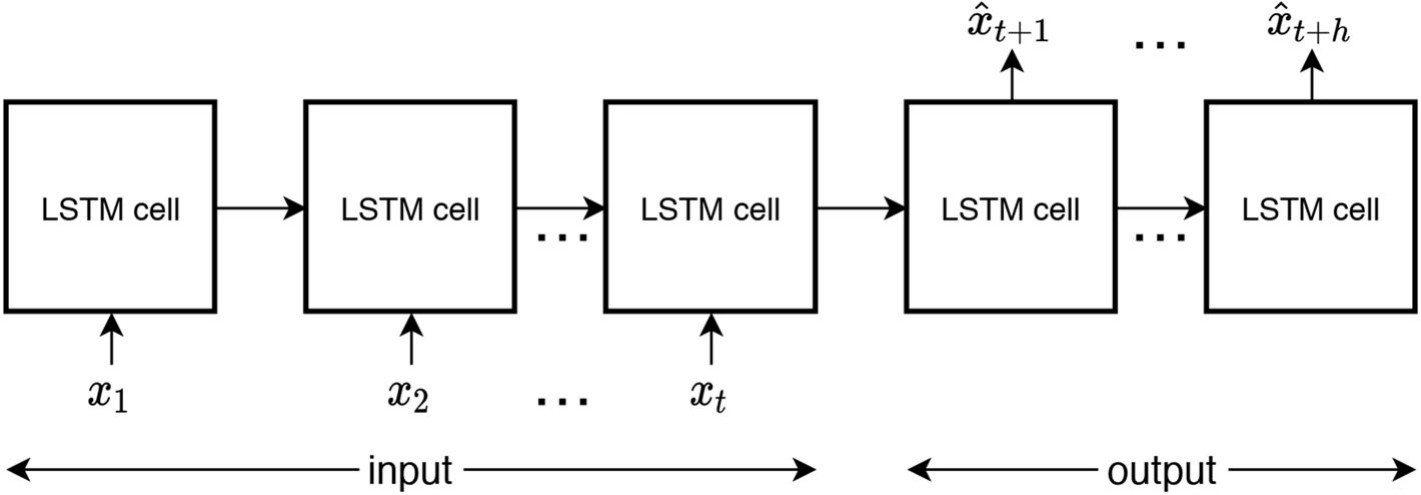
Many to many LSTM network

A LSTM network can be structured in different ways depending on the number of results to be obtained. It can be structured with one input and one output (one to one), many inputs and one output (many to one), one input and many outputs (one to many) or many inputs and many outputs (many to many). Since the fundamental objective of this work is to predict the next h values based on a historical dataset, we are faced with a “many to many” problem, as is depicted in Fig. 1.

Each of the LSTM cells receives the information modeled from the previous cells ($C_{t-1}$ and $h_{t-1}$), as well as the data at the current time instant ($x_t$). Depending on a set of logic gates, it is determined the degree of influence that the data at previous time instants has on data at the time instant to be predicted, thus modeling the behavior of the network.

**Fig. 2:**
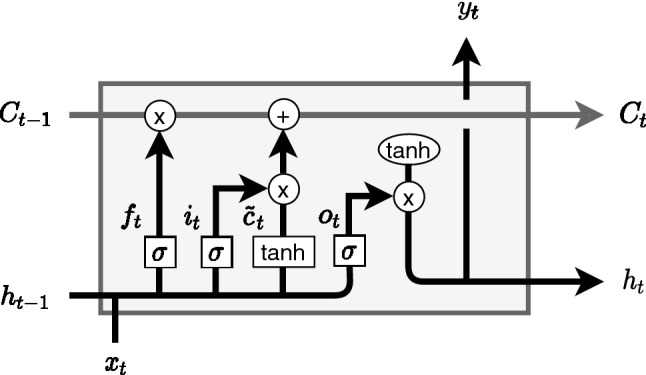
LSTM cell

The scheme of a LSTM cell can be seen in the Fig. 2, where $f_t$ is the forget gate, $i_t$ is the input gate and $o_t$ the output gate. $f_t$ decides what information should be thrown away or saved. A value close to 0 means that the past information is forgotten while a value close to 1 means that it remains. $i_t$ decides what new information $o_t$ to use to update the $c_t$ memory state. Thus, $c_t$ is updated using both $f_t$ and $i_t$. Finally, $o_t$ decides which is the output value that will be the input of the next hidden unit.

The information of the $h_{t-1}$ previous hidden unit and the information of the $x_t$ current input is passed through the $\sigma$ sigmoid activation function to compute all the gate values and through the tanh activation function to compute the $o_t$ new information, which will be used to update. The equations defining a LSTM unit can be summarized as follows:

2 $$\begin{aligned} {\widetilde{c}}_t&= \tanh (W_c[a_{t-1},x_{t}]+b_c) \end{aligned}$$

3 $$\begin{aligned} i_t&= \sigma (W_u[a_{t-1},x_{t}]+b_u) \end{aligned}$$

4 $$\begin{aligned} f_t&= \sigma (W_f[a_{t-1},x_{t}]+b_f) \end{aligned}$$

5 $$\begin{aligned} o_t&= \sigma (W_o[a_{t-1},x_{t}]+b_o) \end{aligned}$$

6 $$\begin{aligned} c_t&= i_t * {\widetilde{c}}_t + f_t * c_{t-1} \end{aligned}$$

7 $$\begin{aligned} a_t&= o_t * \tanh (c_t) \end{aligned}$$

where $W_u$, $W_f$ and $W_o$ and $b_u$, $b_f$ and $b_o$ are the weights and biases that govern the behavior of the $i_t$, $f_t$ and $o_t$ gates, respectively, and $W_c$ and $b_c$ are the weights and bias of the $o_t$ memory cell candidate. An exhaustive description of each of the logic gates, as well as the detailed operation of the LSTM networks can be found in [5].

### Hyperparameter optimization

It is well known that the performance of deep learning models is highly influenced by the choice of the hyper-parameters. This makes a fine-tuning is a determining factor in the training phase to obtain a competitive model. There are several hyper-parameter optimization methods, such as hand-made, grid, random, pseudo-random or probabilistic search, as described in [32]. In this work a hyper-parameter optimization using a random search strategy has been developed using Keras-Tuner framework under Python language [16]. Keras-Tuner is a library developed by the Keras team that contains several hyper-parameters optimization strategies for models developed with Keras and Tensorflow 2.0.

**Fig. 3:**
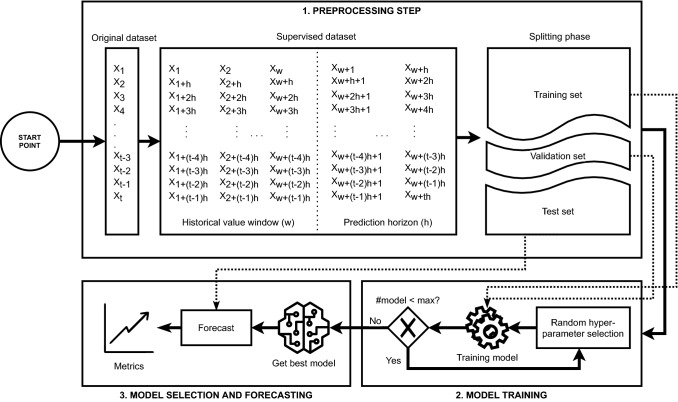
A general overview of the proposed methodology

Depending on a maximum number of trials and the number of models to be trained for each trial, random combinations of all available hyperparameters forming the search space are generated. With each one of these combinations, a model is trained, storing the one with the highest performance as the best model. A complete workflow of the proposed methodology can be seen in Fig. 3.

Additionally, the same deep learning architecture has been optimized using a different hyperparameter optimization strategy. In this case, the heuristic-based model CVOA has been chosen [13]. This algorithm is based on the COVID-19 propagation model and starts with a first infected individual (patient zero), who keeps infecting other individuals, creating large infected populations that will either spread the infection or die. Initially, the infected population grows exponentially, but with factors such as isolation, mortality rate and recoveries, the infected population decreases over time.

## Results

This section reports the results obtained by the proposed LSTM model when optimizing by the two optimization approaches described in Sect. 3.3. First, Sect. 4.1 describes the data set used in this study. Later, the error metrics used to measure the effectiveness of the LSTM model are presented in Sect. 4.2. Finally, Sect. 4.3 analyzes the results obtained, comparing them with other methods published in the literature.

### Dataset description

The time series used in this work is the electricity consumption in Spain from January 2007 to June 2016. It is composed of 9 years and 6 months with a 10-min frequency, resulting in a total of 497832 samples. Based on the results published in previous works [31], the value of *w* has been set to 168, that is, the past values of one whole day and 4 h are used to predict the next 24 values which correspond to the next 4 h. Then, the time series has been transformed into a supervised data set composed of instances and features using the values of the historical window *w* and the prediction horizon *h*, as depicted in Fig. 3. Thus, each instance is made of 192 features, past 168 values and next 24 values. Once this data set has been generated, it has been normalized to the [0,1] range and divided into 70% as training set and 30% as test set. In addition, the training subset has been further divided into 70–30% as a training and validation set to find the optimal values of the hyperparameters of the deep learning model in the model optimization phase. Once the model has been trained and optimized using the training and validation subsets, the test set will be used to check its performance. The training, validation and test sets are composed of 11612, 2903 and 6221 instances, respectively, covering the time periods described in Table 1.

**Table 1:** Distribution of data in training, validation and test sets

Subset	From	To
Training	2007-01-01 00:00	2012-04-23 02:30
Validation	2012-04-23 02:40	2013-08-19 22:40
Test	2013-08-19 22:50	2016-06-21 19:40

### Error metrics

To evaluate the model’s performance, several measures that are widely used in the literature have been used. In particular, the mean absolute error (MAE), mean absolute percentage error (MAPE), the root mean squared error (RMSE) and the mean squared error (MSE) have been chosen as error metrics in this work. The equations defining these metrics are shown below:

8 $$\begin{aligned} \mathrm{MAE} = \frac{1}{n} \sum ^n_{i=1}|p_{i}-a_{i}| \end{aligned}$$

9 $$\begin{aligned} \mathrm{MAPE} = 100 \cdot \frac{1}{n} \sum ^n_{i=1}\frac{|p_{i}-a_{i}|}{a_{i}} \end{aligned}$$

10 $$\begin{aligned} \mathrm{RMSE} = \sqrt{\frac{1}{n} \sum ^n_{i=1}(p_{i}-a_{i})^2} \end{aligned}$$

11 $$\begin{aligned} \mathrm{MSE} = \frac{1}{n} \sum ^n_{i=1}(p_{i}-a_{i})^2 \end{aligned}$$

where *n* is the number of samples to be predicted and, $p_i$ and $a_i$ are the predicted and actual values of the *i*-th sample, respectively.

### Experimental setup and analysis

This section reports the results of the training of the proposed LSTM model and the process of searching for the best values of the hyperparameters using a random search and the CVOA heuristic search strategy along with forecasts obtained for the test set. Furthermore, it is compared with a TFT model, as well as with other models recently published in the literature.

The experiments have been run in a Intel Core i7-5820K at 3.3 GHz with 15 Mb of cache, 6 cores with 12 threads, 64 GB of RAM memory and a Nvidia Titan V GPU, working under Ubuntu 18.04 operating system.

One of the main questions is whether it is feasible to optimize all the hyperparameters of the network. For example, several ad-hoc estimation methods are known in the literature for some hyperparameters such as the learning rate, thus avoiding their optimization in the training phase. In this work, a first approach based on callbacks is proposed, such that the learning rate is dynamically adjusted after several iterations without a significant margin of improvement, a value that must be established in advance. A LSTM architecture was trained with a total of 500 epochs applying variable learning rate. This network obtained a MAPE of more than 10%. The same network was tested optimizing the learning rate in the training phase. The results were better, and for this reason, it was decided to add the learning rate as a further hyperparameter to be optimized.

Table 2 presents the hyper-parameters that have been optimized in this work. In addition, it shows the minimum and maximum values and the step of increase established for each of the parameters. Dropout and learning rates do not include steps because they do not follow any criteria, but they are randomly generated numbers between the minimum and maximum value for the dropout rate and among the discrete values $\{0.1, 0.01, 0.001, 0.0001, 0\}$ for the learning rate.

**Table 2:** Hyper-parameter search space for two optimization method

Parameter	Random			CVOA		
	Min.	Max.	Step	Min.	Max.	Step
Hidden layers	1	10	1	1	12	1
Units per layer	50	300	25	25	300	25
Dropout rate	0	0.4	–	0	0.45	–
Learning rate	0.0001	0.1	–	0	0.1	–

In Keras-Tuner, the maximum number of trials has been set to 10 and a maximum of 20 models for each trial. Thus, a total of 200 models are trained in order to obtain the best hyperparameters, and therefore the optimal network architecture for the proposed deep LSTM. To reduce the training time, a total of 30 periods and a lot size of 256 are used in the optimization phase. Once the best of all models has been obtained, the model is retrained with a total of 500 epochs. The model has been trained using the MAE metric as the loss function.

In the CVOA approach, a total of 10 iterations have been established. Due to the nature of the method, the number of models tested for each iteration grows exponentially. Thus, a total of 973 models have been tested. In order to minimize the execution time, the optimization was performed on a reduced subset with a total of 30 epochs. Once the best model of this optimization was obtained, it was re-trained with a total of 300 epochs and a batch size of 256.

The network architectures of the two best models obtained by the random and the CVOA search strategy are summarized in Tables 3 and 4, respectively.

**Table 3:** Architecture of the best LSTM model using random search

Layer (type)	Number of units	Number of parameters
LSTM	75	23,100
LSTM#1	200	220,800
Dropout#1	200	–
LSTM#2	275	523,600
Dropout#2	275	–
LSTM#3	225	450,900
Dropout#3	225	–
Dense	24	5424

For the random search, the optimal deep learning model is composed of a total of five layers: the input layer, three hidden layers and the output layer. The input layer and the three hidden layers are LSTM layers and the output layer is one dense layer. The optimal dropout rates are applied on hidden layers in order to avoid overfitting of the deep LSTM network in its training. The optimal value of the learning rate is 0.001. The input layer consists of 75 recurrent units and receives information from the training set. A layer with 200 recurrent units is applied again on this output. Once the first hidden layer has been applied, a dropout rate of 0.4 is used, which implies randomly discarding 40% of the recurrent cells. Once 40% of the neurons have been discarded, the process is repeated with the second and third LSTM hidden layers, where 275 recurrent units with a dropout rate of 0.3 and 225 recurrent units with a dropout rate of 0.2 are used, respectively. Finally, a dense layer is applied to obtain the 24 values of the prediction horizon ($h=24$) as output of the network.

**Table 4:** Architecture of the best LSTM model using random search

Layer (type)	Number of units	Number of parameters
LSTM	175	123,900
LSTM#1	200	300,800
LSTM#2	25	22,600
LSTM#3	225	225,900
LSTM#4	175	280,700
LSTM#5	125	150,500
LSTM#6	225	315,900
LSTM#7	300	631,200
Dense	24	7224

For the CVOA method, the best model obtained is composed of seven layers, all of them recurrent layers except for the last one. The last layer corresponds to a dense layer, used to provide the expected output. In this model, no dropout rate is applied, so none of the neurons computed throughout the network architecture are discarded. The optimal value of the learning rate is 0.0001. This model requires training more than two million parameters, thus implying a high computational cost.

Table 5 shows the prediction errors in terms of MAE, MAPE and RMSE obtained by the best LSTM model for the test set using the two optimization strategies. It can be seen that relative errors below 1.5% are reported, showing the effectiveness of the LSTM to predict Spanish electricity demand time series.

**Table 5:** Prediction errors obtained by the LSTM for the test set

Metric	LSTM+Random	LSTM+CVOA
MAE (MW)	398.7652	435.9883
MAPE (%)	1.4472	1.5898
RMSE (MW)	545.8998	585.1958

As can be seen in Table 5, the random strategy achieves better results than the heuristic-based approach, so the following analysis of results will focus on the random hyperparameter optimization.

**Fig. 4:**
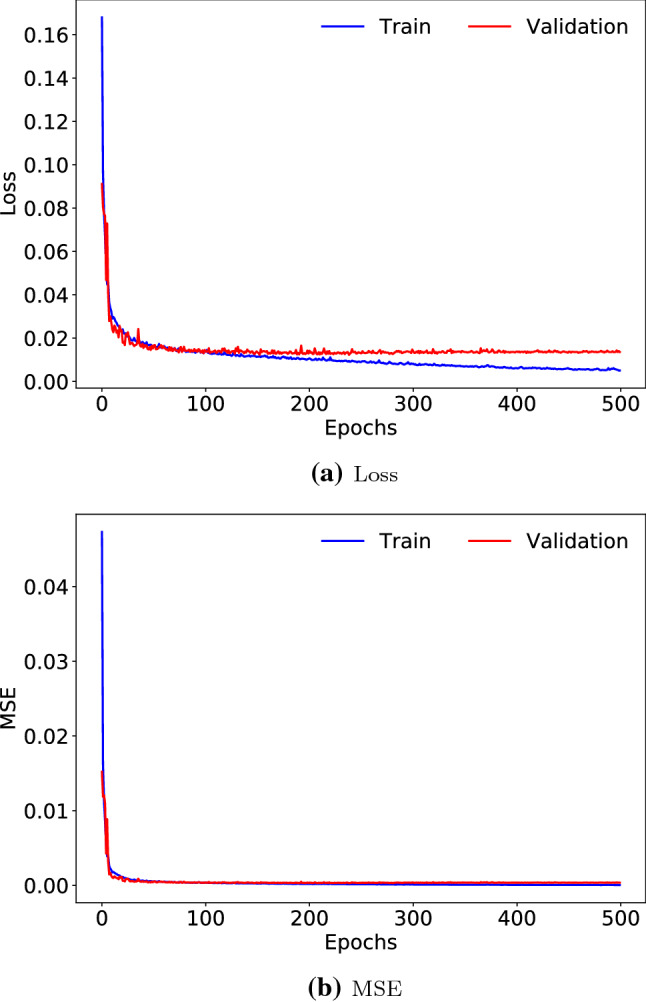
Evolution of the model training through 500 epochs

Figure 4 shows the evolution of the best model over 500 epochs in the training process. In particular, the MAE loss function and the MSE metric for the training and validation sets are presented. In the training phase, it can be observed how the MAE and MSE decrease as the number of epochs increases, thus showing the convergence of the model and the absence of overfitting. A typical sign of overfitting is shown when train loss is going down, but validation loss is rising. However, Fig. 4a does not present this behavior, since both train loss and validation loss decrease as the epochs increase. Furthermore, it can be observed that the loss function stabilizes in the validation set in the later epochs, so it can be interpreted that the model will not improve significantly if the number of epochs is further increased.

**Fig. 5:**
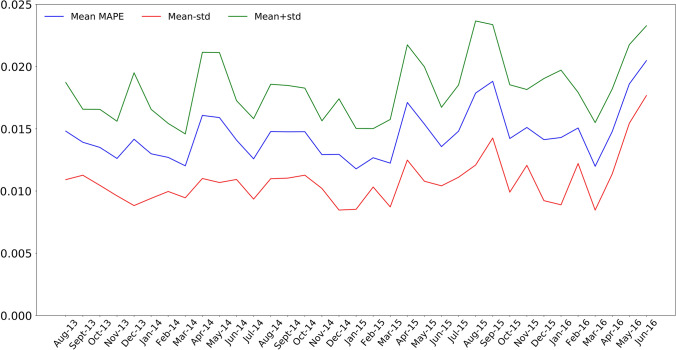
MAPE along with the standard deviation for each month of the test set

**Fig. 6:**
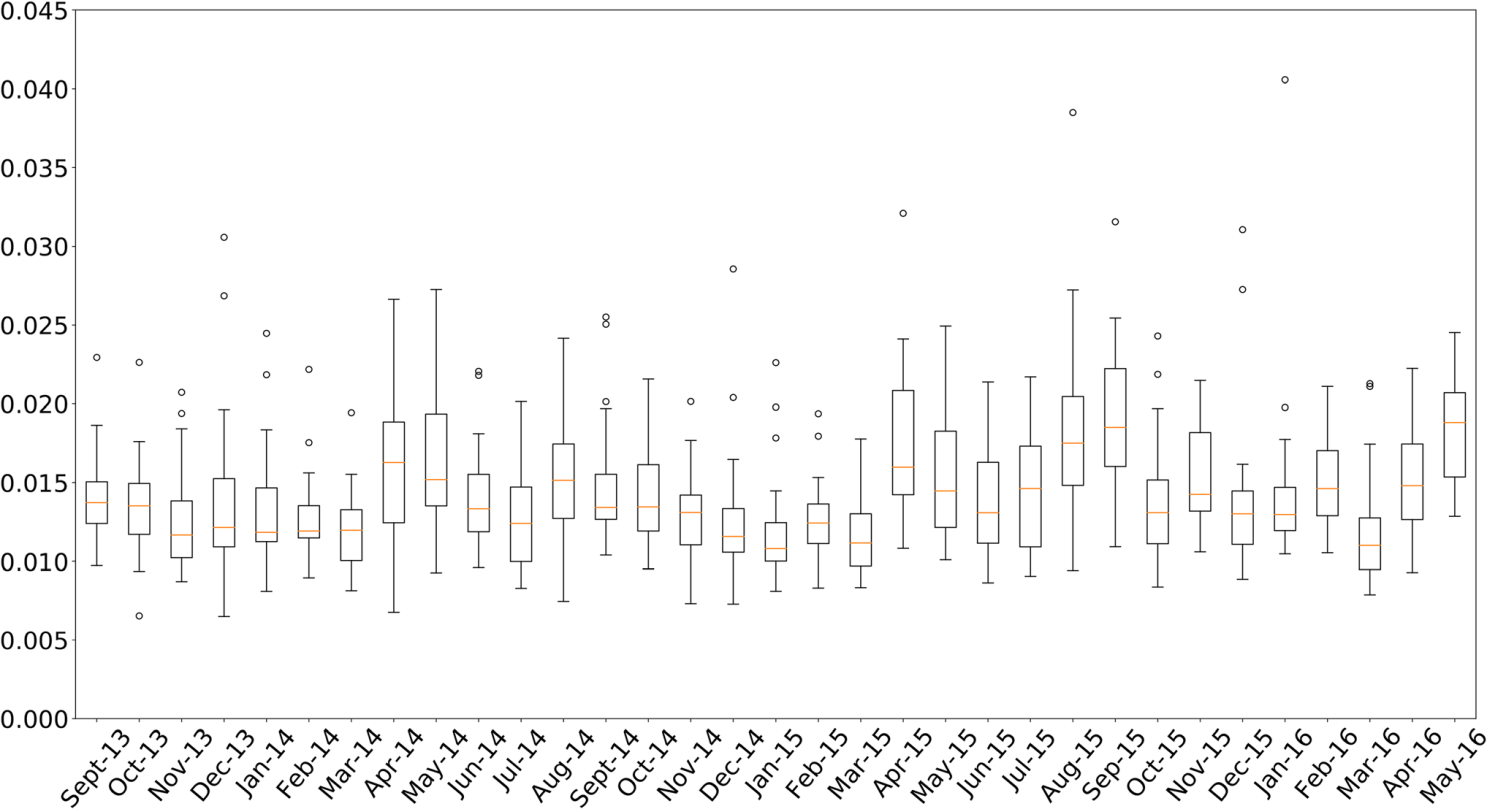
Distribution of the MAPE values for each month of the test set

From Figures 5 and 6, the uncertainty of the predictions obtained by the best LSTM can be analyzed. Figure 5 presents the monthly average of the MAPE and standard deviations of the predictions for months of the test set, that is, from September 2013 to May 2016. The average of the standard deviation for these months is 3.7%, reaching the highest deviation of 5.8% in the month of August 2015. Figure 6 presents the variability of MAPE values for the months of the test set. It can be seen that there are very few outliers errors and they do not represent a significant number as 75% of all errors in every month are below 2%. The months of greatest uncertainty correspond to the months of April and May, which belong to the spring season, which is a very unstable season from a meteorological point of view, and August, which is atypical due to the fact that it is a common vacation month in Spain.

**Fig. 7:**
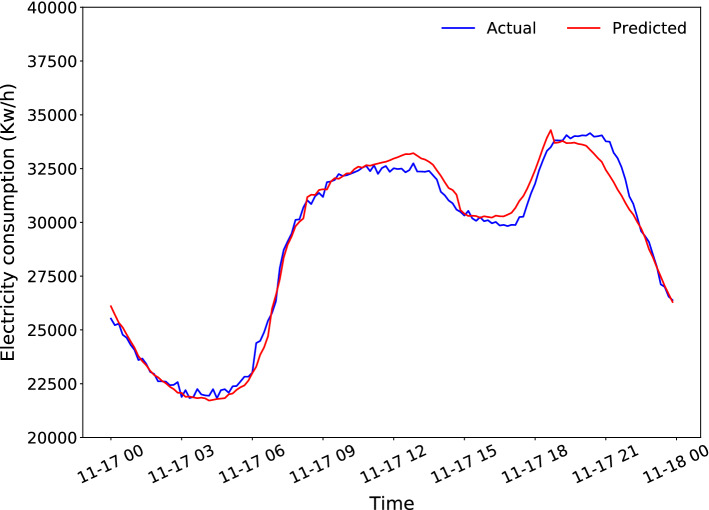
Best daily prediction

**Fig. 8:**
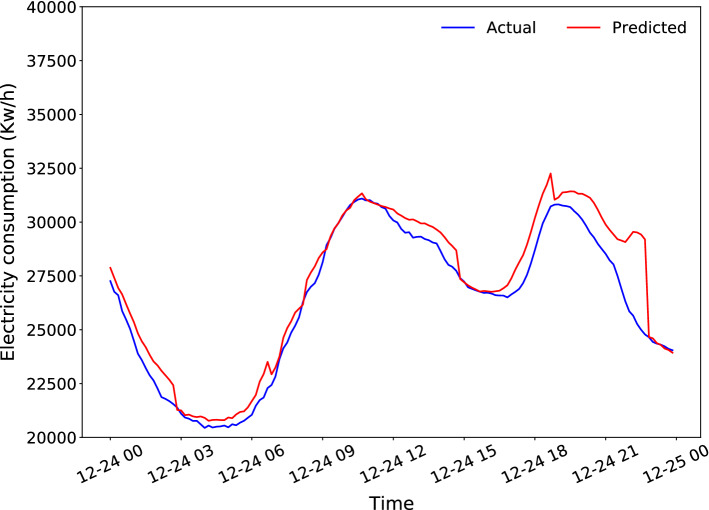
Worst daily prediction

Figures 7 and 8 present the best and worst days predicted by the proposed LSTM model, achieving a MAE of 0.1990 MW and 730.5677 MW, respectively. The best day corresponds to November 17, 2015, while the worst day corresponds to December 24, 2014, which is a day marked on the calendar as Christmas Eve. Moreover, the greatest error is made at the end of the day, which corresponds to the time slot where the celebration of that day is usual.

**Fig. 9:**
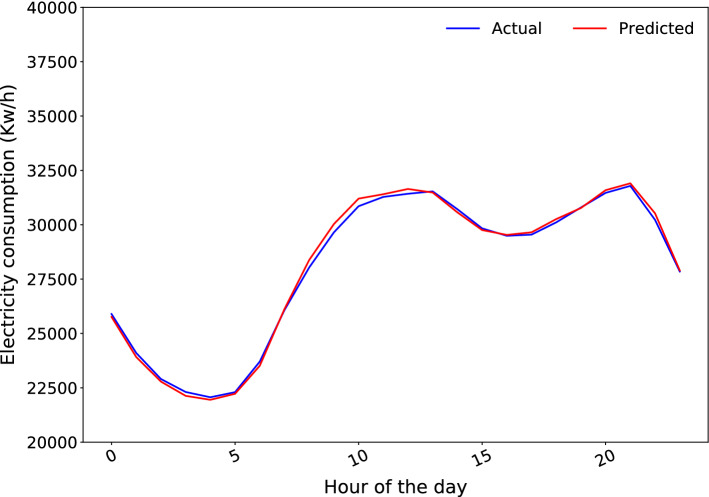
Hourly average of the predictions for test set

Figure 9 shows the hourly average of the forecasts obtained by the LSTM when predicting the test set. It can be seen that the model fits extremely well at all times of the day.

**Fig. 10:**
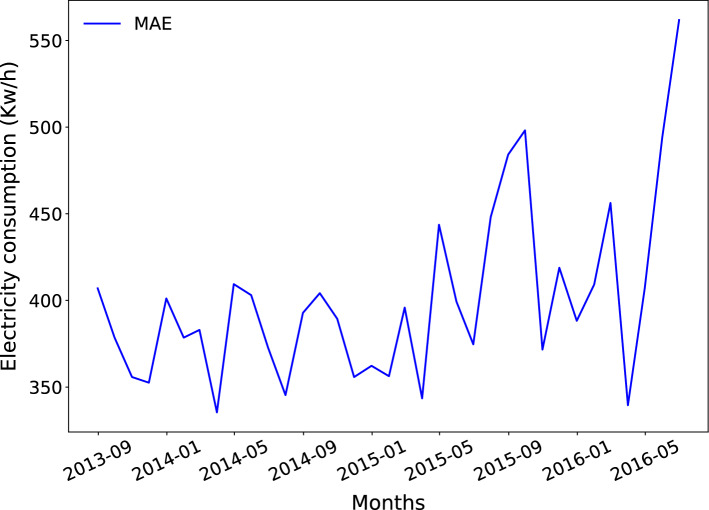
Monthly average of the absolute errors for the test set

Figure 10 presents the average of the absolute errors for all months of the test set. It can be seen that the worst predicted months correspond to June 2016 and September 2015 with a MAE of 561.8191 MW and 498.1631 MW, respectively.

In order to evaluate the performance of the LSTM, the errors have been compared with those of other forecasting methods published in [31]. In particular, a linear regression (LR) as the state-of-art reference model, a regression tree (DT) based on a greedy algorithm, two well-known ensembles of trees such as gradient-boosted trees (GBT) and random forest (RF) and a single-output deep feed-forward neural network (DFFNN) for each value of the prediction horizon. The optimal values of the hyperparameters obtained by a grid search in [15] have been used for each of these methods. Specifically, a learning rate of $1E-10$ and 100 iterations for the gradient descent method in the LR, one tree of depth 8 for DT, 5 and 100 trees of depth 8 for GBT and RF, respectively, and layers ranging from 2 to 5, and neurons between 40 and 100 for each DFFNN. In the case of the TFT network, the learning rate was set at 0.0794 and the hidden size and the hidden continuous size were set to 32 and 8, respectively. Other parameters that were also optimized are the attention head size and the number of lstm layers, which were set to 2 and 1, correspondingly.

Table 6 shows the MAPE obtained by the LSTM, and the above benchmark methods when predicting the test set. It can be seen that the proposed LSTM using the random search significantly improves the MAPE obtained by the other forecasting models.

**Table 6:** MAPE obtained by the proposed LSTM, TFT, DFFN and other machine learning methods

	MAPE (%)
LR	7.3395
DT	2.8783
GBT	2.7190
RF	2.2005
DFFN	1.6769
LSTM+CVOA	1.5898
TFT	1.5148
LSTM+Random	1.4472

## Conclusions

In this work, a deep neural network has been designed specifically to predict electricity demand time series. A LSTM network architecture has been proposed due to its ability to deal with sequential data as its main characteristic is memory for retaining temporal relationships in the long term. A random search and the CVOA metaheuristic to find the best values of the hyperparameters such as number of layers, number of LSTM cells for layer, learning and dropout rates have been carried out. Once these optimal values are determined, the best LSTM network is applied to the Spanish electricity demand from 2007 to 2016 with a 10-min frequency to obtain forecasts for the next 24 values. Results report very accurate predictions reaching errors of less than 1.5%. In addition, the proposed LSTM network has obtained the smallest errors when compared with a linear regression, a decision tree, two ensembles of trees and two deep neural networks such as a deep feed-forward neural network optimized using a random search and a TFT optimized using a sampling algorithm.

Future work will be directed towards the fusion of different deep learning models to exploit the different advantages of each model in order to obtain predictions for different real-world problems.
